# Two Different Brain Injury Patterns Associated with Compound Heterozygosis of the PIGO Gene in a Term Newborn: A Case Report

**DOI:** 10.3390/biomedicines12122779

**Published:** 2024-12-06

**Authors:** Francesco Dellepiane, Giulia Moltoni, Sara Ronci, Alessia Guarnera, Maria Camilla Rossi-Espagnet, Maria Cristina Digilio, Diego Martinelli, Francesca Campi, Daniela Longo

**Affiliations:** 1 Diagnostic and Interventional Neuroradiology Unit, Bambino Gesù Children’s Hospital, IRCCS, 00165 Rome, Italy; giulia.moltoni@uniroma1.it (G.M.); guarneraalessia@gmail.com (A.G.); mcamilla.rossi@opbg.net (M.C.R.-E.); daniela.longo@opbg.net (D.L.); 2 Neuroradiology Unit, NESMOS S. Andrea Hospital, University Sapienza, 00189 Rome, Italy; 3 Neonatal Intensive Care Unit, Bambino Gesù Children’s Hospital, IRCCS, 00165 Rome, Italy; sara.ronci@opbg.net (S.R.); francesca.campi@opbg.net (F.C.); 4 Medical Genetics Unit, Bambino Gesù Children’s Hospital, IRCCS, 00165 Rome, Italy; mcristina.digilio@opbg.net; 5 Metabolic Diseases Unit, Bambino Gesù Children’s Hospital, IRCCS, 00165 Rome, Italy; diego.martinelli@opbg.net

**Keywords:** PIGO, gene, mutation, compound heterozygosis, bran MRI, newborn

## Abstract

The glycosylphosphatidylinositol (GPI) is a glycol–lipid that anchors several proteins to the cell surface. The GPI-anchor pathway is crucial for the correct function of proteins involved in cell function, and it is fundamental in early neurogenesis and neural development. The PIG gene family is a group of genes involved in this pathway with six genes identified so far, and defects in these genes are associated with a rare inborn metabolic disorder manifesting with a spectrum of clinical phenotypes in newborns and children. Among them, the PIGO gene encodes for phosphatidylinositol glycan anchor biosynthesis class O protein (PIGO), an enzyme participating in this cascade, and the loss of its function often leads to a severe clinical picture characterized by global developmental delay, seizures, Hirschsprung disease, and other congenital malformations. To date, 19 patients with confirmed PIGO deficiency have been described in the literature with a host of clinical and radiological manifestations. We report a case of a male term newborn with two compound heterozygous variants of the PIGO genes, presenting with encephalopathy, drug-resistant epilepsy, and gastrointestinal abnormalities. Brain MRI first showed diffusion restriction in the ponto-medullary tegmentum, ventral mesencephalon, superior cerebellar peduncles, cerebral peduncles, and globi pallidi. This pattern of lesion distribution has been described as part of the neuroradiological spectrum of PIG genes-related disorders. However, after one month of life, he also showed a previously undescribed MRI pattern characterized by extensive cortical and subcortical involvement of the brain hemispheres. The presence of two different mutations in both the PIGO genes may have been responsible for the particularly severe clinical picture and worse outcome, leading to the death of the newborn in the sixth month of life despite therapeutic attempts. This case expands the neuroradiological spectrum and may bring new insights on glycosylation-related disorders brain manifestations.

## 1. Introduction

The glycosylphosphatidylinositol (GPI) is a glycol–lipid that anchors more than 150 different cell surface proteins. Therefore, the glycosylphosphatidylinositol anchor pathway (GPI-AP) is crucial for the post-translational modification of numerous proteins vital for cell signaling, and it is fundamental to early human neurogenesis and neural development. The biosynthesis of the GPI anchor and its attachment to the proteins require a complex cascade of more than 20 gene products, and defects in this cascade lead to rare inborn metabolic disorders. The glycosylphosphatidylinositol (GPI)-anchor biosynthesis pathway begins at the level of the endoplasmic reticulum (ER), where a phosphatidylinositol group is inserted into the membrane with the subsequent composition of a glycan core composed of glucosamine and mannose, and it is decorated with phosphoethanolamine moieties to which the proteins attach. The GPI-anchored protein complex is then transferred from the ER to the Golgi apparatus, where it undergoes remodeling. The GPI-anchored remodeled proteins are then trafficked to the outer side of the plasma membrane, where they become part of the lipid raft domains. More than 150 proteins are known to be associated with lipid rafts via a GPI anchor, including adhesion proteins and extracellular receptors, enzymes, immune components, and others, and they are vital to several cellular functions.

The PIG (phosphatidylinositol glycan anchor biosynthesis) gene family is a group of genes involved in the GPI-anchor pathway. So far, six genes (PIGA, PIGL, PIGM, PIGN, PIGO, and PIGV) have been identified, and patients with mutations of these genes are likely to present symptoms associated with disorders of glycosylation, often presenting with severe neurological manifestations such as seizures, muscular hypotonia, and intellectual disability, which is associated with a spectrum of other disorders including facial dysmorphisms and severe gastrointestinal abnormalities.

The PIGO gene encodes for phosphatidylinositol glycan anchor biosynthesis class O protein (PIGO), which is an enzyme responsible for attaching the terminal phosphoethanolamine residue to the third mannose residue of the GPI glycan core [1]. Loss of its function leads to a severe clinical picture characterized by global developmental delay, seizures, Hirschsprung disease, and other congenital malformations [2].

The exact etiopathogenesis of symptoms in PIGO deficit is still not clarified. Regarding the central nervous system, researchers have observed a substantially reduced myelination as well as a pronounced astrogliosis and microgliosis of the white matter in patient with PIGO mutations [3]. The theory of myelination damage was supported also in a mouse model study, showing a decrease of 70%, compared to control levels, of myelin basic protein in PIGO-mutated mouse brain [4].

To date, 19 patients with confirmed PIGO deficiency have been described in the literature. We present a new case of PIGO deficiency caused by previously unreported compound heterozygous PIGO variants and displaying a severe neurological and gastrointestinal phenotype.

## 2. Case Presentation

A male newborn 18 h old, who was the third child of Nigerian parents and born at 38 weeks of gestational age via cesarian section, was admitted to our institution for abdominal distension and dysmorphic features with brachydactyly, hypoplasia of the distal part of fingers and nails, feet with small and dysplastic nails, peculiar facies characterized by elongated palpebral fissures and bilateral preauricular pits.

No complications were reported during the pregnancy or at birth, but no information about prenatal screening and ultrasound was available. TORCH screening, as well as vaginal and rectal swabs, were negative. Nothing was to report in the family history.

Brain ultrasound (US) investigations were carried out regularly without any significant finding. Abdomen US performed showed no abnormalities in the kidneys, spleen and liver but revealed marked overdistention of the intestinal loops by corpuscular fluid material with thinned walls. Due to abdominal distension and difficulty in spontaneous evacuation, more radiological investigations were performed, including abdomen X-rays confirming bowel dilatation and an abdomen barium enema, revealing altered intestinal transit with reduced distensibility of the colon.

On the third day of life, an exploratory laparotomy was performed, and a low ileostomy was made. Intestinal biopsies, which were performed at the same time, led to the diagnosis of congenital aganglionic megacolon.

Subsequently, due to the non-functioning stomia causing difficulty in evacuation and the impossibility of feeding the newborn per os because of poor suction, a more proximal ileostomy was remade and a gastrostomy was positioned at 30 days of life. However, the newborn continued to show poor feeding tolerance and a high ostomy output enough to cause significant electrolytic losses and dehydration. Therefore, it was necessary to continue parenteral nutrition via a central venous catheter (CVC). Blood tests at admission showed no abnormalities except for an increase in alkaline phosphate (ALP) (1689 U/L, n.v. 140–500 U/L) and hyperphosphatasia (7.3 g/dL).

At one week of age, the neonate began to present persistent tremors spreading to the limbs, eyes and trunk associated with desaturation. An electroencephalographic (EEG) study was performed and did not show a clear electrical correlate but highlighted a highly immature tracing for the age. Brainstem auditory evoked potentials were absent. A brain ultrasound showed no parenchymal anomalies. Due to the persistence of tremors despite medical therapy, at 12 days of life, a brain magnetic resonance imaging (MRI) was performed, showing brainstem alterations.

First and second-level metabolic screenings were performed, testing plasma amino acids, urinary organic acids and acylcarnitines, yet no abnormalities were found; genetic investigations in trios were also started. The newborn was also subjected to an extensive neurophysiological study with evidence of normal visual evoked potentials and electroretinography but absent somatosensory evoked potentials. Despite maximal therapy with pyridoxine, baclofen, lorazepam, gabapentin and clonazepam, tremors associated with generalized hypertonicity progressively worsened, making non-invasive respiratory support necessary.

A second brain MRI, repeated at about one month of life, showed a worsening evolution of the neuroradiological findings (both first and second MRI characteristics are described in the dedicated section). Genetic tests revealed a compound heterozygosis of the PIGO gene. After communicating the results of the genetic tests to the parents and after discussing with them the unfavorable prognosis of the disease, the Ethics Committee (EC) of our hospital was involved in future therapeutic decisions in order to identify the best care context for the baby. The EC suggested to abstain from invasive diagnostic-therapeutic procedures that were unable to modify the clinical course of the baby and to implement only those aimed at reducing and alleviating the pain. Consequently, the baby was gradually weaned from oxygen, reaching satisfactory saturation levels. The start of an analgesic-sedation program, which reduced the painful crises that were not responsive to the therapy administered so far, was agreed upon.

The baby died at 6 months of age.

### 2.1. MRI Features

The baby was subjected to three subsequent MRI examinations with the “feed and wrap” technique for neonatal imaging, respectively at 15 days, 35 days, and 3 months of life.

Both a 3T scanner (Siemens MAGNETOM Skyra ©, Siemens Healthcare, Erlangen, Germany) and a 1.5T scanner (MAGNETOM Aera ©, Siemens Healthcare, Erlangen, Germany) were used depending on availability.

Scanning protocol always included axial T1-weighted spin–echo sequences, axial and coronal T2-weighted turbo-spin–echo sequences, 3D magnetization prepared rapid gradient echo imaging (MPRAGE) sequences, axial diffusion-weighted imaging (DWI) and related apparent diffusion coefficient (ADC) maps, and susceptibility weighted imaging (SWI); in the second and third exams, a single-voxel MR spectroscopy with a TE of 30 ms was also performed in the site of the brain alterations. None of the examinations required the injection of gadolinium-based contrast medium.

**Figure 1:**
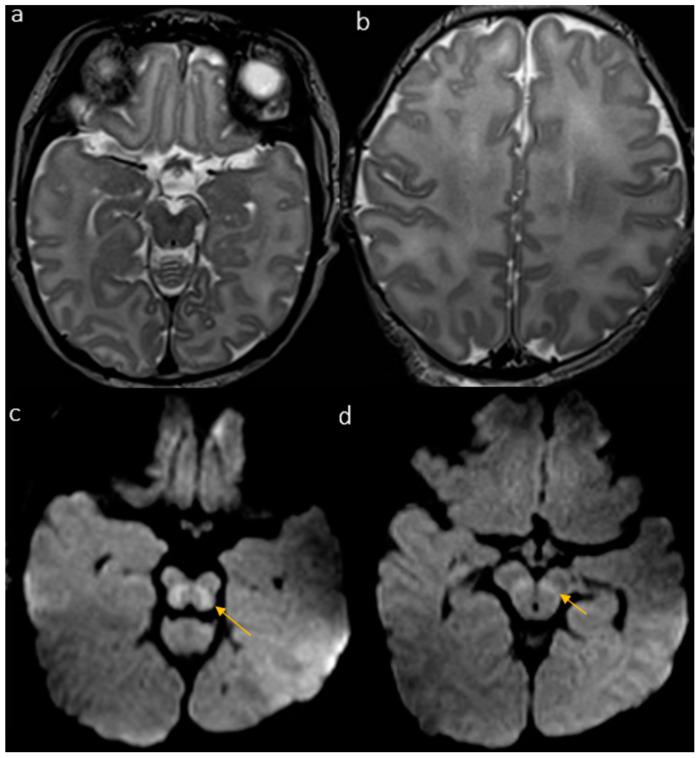
The first MRI at 15 days of life shows a “mild” pattern with no evident abnormalities of the brain cortex and WM (**a**,**b**) but diffusion restriction of the midbrain tegmentum (**c**) and cerebral peduncles (**d**) on DWI (arrows).

The first brain MRI showed bilateral symmetrical diffusion restriction on DWI/ADC of the superior cerebellar peduncles, the dorsal mesencephalus, the dorsal aspect of the cerebral peduncles and the globi pallidi (Figure 1). The remaining brain findings were nonspecific and included corpus callosum thinning and an increased T2WI signal at the level of the periventricular white matter. These first findings we classified as a “mild” neuroradiological pattern of brain involvement, and it has been described in the previous literature as part of the neuroradiological spectrum of PIG-related encephalopathy.

**Figure 2:**
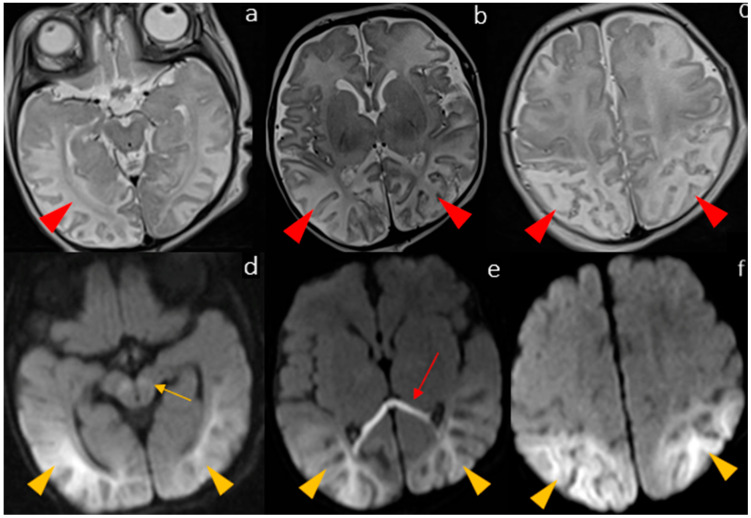
The MRI performed at 35 days shows a “severe” pattern with diffuse T2 hyperintensity of the brain cortex and WM, which is prevalent in the parieto-temporo-occipital lobes ((**a**–**c**), red arrowheads), pairing with a significant diffusion restriction in the same regions ((**d**–**f**), yellow arrowheads). There is also diffusion restriction of the corpus callosum, consistent with pre-Wallerian degeneration ((**e**), red arrow), and persistent diffusion restriction in the midbrain ((**d**), yellow arrow).

The second MRI (Figure 2) confirmed the previous alterations but also showed extensive cortical and subcortical white matter T2WI hyperintensity and diffusion restriction on DWI, mostly located in the parietal and temporal regions of brain hemispheres, and in the splenium of the corpus callosum (this last finding was probably subsequent to pre-Wallerian degeneration). These second findings we called a “severe” pattern with more extensive brain involvement that has not been described in the previous literature, and it led to diffuse gliotic-malacic changes with multicystic degeneration and shrinkage of the parietotemporal cortex as well as adjacent white matter (sites of the previous alterations).

**Figure 3:**
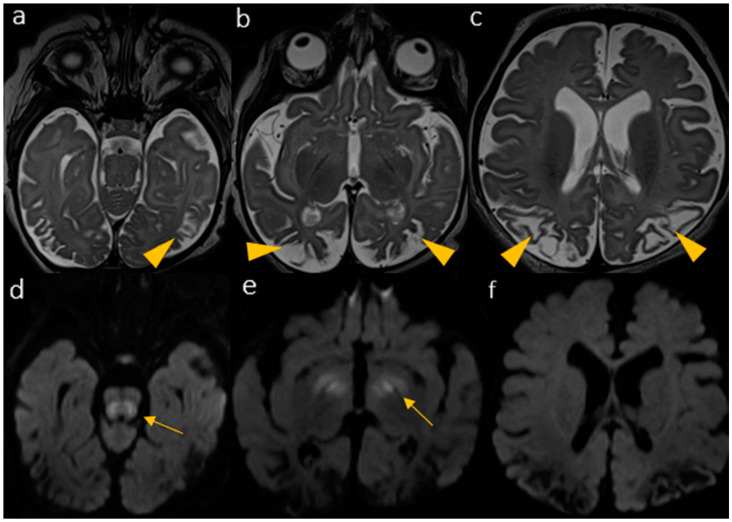
The last MRI, performed after an additional 2 months, shows overall brain tissue shrinkage and ventricle/subarachnoid space dilatation, cystic and gliotic degeneration of the WM, particularly in the previously involved parieto-temporo-occipital lobes characterized by T2 hyperintensity ((**a**–**c**), arrowheads) with resolution of diffusion restriction on DWI (**f**). There is persistent diffusion restriction of the midbrain tegmentum (**d**) and basal ganglia (**e**) (arrows).

A third follow-up MRI, performed after two additional months (Figure 3), confirmed this evolution.

Interestingly, the diffusion restriction on DWI in the midbrain, globi pallidi, cerebral peduncles and superior cerebellar peduncles (which we considered to be the milder cerebral manifestation of the disease in our case) remained essentially stable through all three examinations. On the other hand, the diffuse cortical and subcortical involvement (which we considered to be the more severe manifestation) had a phase of acute damage and a chronic phase characterized by gliotic-malacic changes and brain tissue atrophy.

### 2.2. Genetic Workup

Molecular analysis was performed with an in silico gene panel, using the NGS sequencing technique performed in trio with a 4 bases ClinEX pro kit on the NovaSeq6000 platform (Illumina ©, San Diego, CA, USA).

The analysis in the context of epileptic encephalopathies identified two compound heterozygous variants (c.839T>C; c.2285C>T) in the PIGO gene, which were associated with hyperphosphatasia syndrome and intellectual disability type 2 (OMIM 614749). At the protein level, these variants determine the amino acid changes p.Met280Thr and p.Pro762Leu, respectively. The missense variant p.Met280Thr, with paternal segregation, which has an allelic frequency of 0.00000398 in the general population (gnomAD), has been described in the scientific literature, has been annotated in the ClinVar database, and can be classified according to the ACMG guidelines as a probably pathogenic variant (class 4). The missense variant p.Pro762Leu, with maternally segregated alleles, which has an allelic frequency of 0.00002 in the general population (gnomAD), has not been described in the scientific literature. It is annotated in the ClinVar database (ID: 1045910) and is classified as a variant of uncertain significance VUS (class 3), i.e., with undefined functional and clinical effects.

The clinical picture characterized by brachydactyly, nail bed hypoplasia, Hirschsprung disease, hypotonia, and epileptiform alterations associated with biochemical alterations such as increased ALP and hyperphosphatasia supported the molecular diagnosis of PIGO defect and defects associated with the synthesis of GPI-anchored glycoproteins: hyperphosphatasia syndrome and intellectual disability type 2 [1].

PIGO deficiency has a broad spectrum of clinical manifestations, which vary from mild phenotypes to severe forms lethal in early childhood.

The severity of symptoms has been shown to be related to the residual activity of the mutant PIGO [5]. In our patient, both mutations were missense, thus giving rise to a truncated and non-functional protein. This may justify the phenotypic severity characterized by intractable epilepsy and developmental delay, total colonic aganglionosis, brachydactyly, and facial dysmorphism as well as the severity and rapid progression of the MRI findings.

## 3. Discussion

The PIGO gene is part of a group of genes involved in the GPI biosynthesis, encoding for phosphatidylinositol glycan anchor biosynthesis class O protein. Both somatic and germline mutations of the PIG family genes have been described in the literature with a vast spectrum varying to mild to severe clinical manifestations.

Among them, somatic mutations of the PIGA gene have been reported in patients with paroxysmal nocturnal hemoglobinuria (PNH) [6,7,8], while germline mutations have long been considered to be lethal. Only recently, germline mutations have been described in male patients with XLIDD (X-linked intellectual developmental disorder) with a wide spectrum of clinical presentations [9]. Also recently, a new missense PIGA germline mutation was described in a Chinese male infant presenting with developmental arrest, infantile spasms, brain MRI alterations, contractures, dysmorphism, elevated alkaline phosphatase, mixed hearing loss, liver dysfunction, mitochondrial complex I and V deficiency, and therapy-responsive dyslipidemia [8].

Regarding the PIGO gene, several case reports have described mutations associated with epileptic encephalopathy both with and without an increase in blood alkaline phosphatase levels [10,11,12]. PIGO mutations are also related to hyperphosphatasia with mental retardation syndrome [13], which is also known as “Mabry Syndrome”. Its first description dates back to 2010 by Thompson et al. [14]. They gave this name to a spectrum of anomalies characterized by dysmorphic features, digital abnormalities, neurodevelopmental abnormalities and persistent hyperphosphatasia. This phenotype was described by Mabry et al. in 1970 [15].

Due to the often nuanced and overlapping patterns in neonatal neurological pathologies, it is sometimes difficult to reach a definitive diagnosis in a short time, and multiple diagnostic and therapeutic efforts are made.

Many patients affected by neurological pathologies with neonatal onset present clinical characteristics such as convulsions, developmental delay and dysmorphism, which however are not sufficient to identify a specific syndromic picture. However, there are laboratory findings that may suggest a specific condition such as, as in our case, an abnormal level of plasmatic ALP. In these cases, it is mandatory to carry out a search for GPI biosynthesis deficiency in order to establish a correct therapeutic approach aimed at managing the important clinical symptoms that this may entail.

In particular, the nutritional management and control of neurological symptoms were the two main clinical challenges in our patient. Despite the gradual reduction up to the suspension of enteral nutrition, the stoma output remained higher than what is typically observed in patients with an ileostomy. As hypothesized by Starosta et al. [2], the intestinal mucosa of these patients may be characterized by a deficit in absorption capacity across all segments due to changes associated with the glycocalyx on the surface of enterocytes. This glycocalyx dysfunction was previously suggested by Brucker et al. [16] in a cohort of patients with glycosylation disorders. We believe that further studies are needed to support this hypothesis and, more importantly, to identify the best nutritional strategy for these patients.

The underlying cause of the neurological symptoms in PIGO dysfunction is a reduced activity of GPI-anchored alkaline phosphatases, especially tissue nonspecific alkaline phosphatase (TNAP). Among its various functions, TNAP is involved in the conversion of the hydrophilic form of vitamin B6, pyridoxal-5’-phosphate, into pyridoxal, which can cross the blood–brain barrier into the central nervous system [17,18]. Here, pyridoxal is involved in the synthesis of γ-aminobutyric acid (GABA), which is one of the main inhibitory neurotransmitters.

However, in our patient, despite the administration of pyridoxine, baclofen, lorazepam, gabapentin, and clonazepam, the neurological symptoms were difficult to control, requiring profound analgesic sedation and high doses of continuous morphine iv infusion.

Neuroradiological manifestations of PIG family genes mutations have also been described in the literature. Zehavi et al. described a case of omozygous PIGO gene mutation causing severe epileptic encephalopathy and corpus callosum hypoplasia in two siblings [12]. Tarailo-Graovac et al. described MRI findings associated with PIGA deficiency, which include reduced myelination, white matter and deep gray matter abnormalities, thinning of the corpus callosum, and cerebral atrophy [9]. Cerebral atrophy tends to progress rapidly and is consistent with an early neurodegenerative process [19]. However, the most specific MRI alterations described by the authors was restricted diffusion with T2WI hyperintensity involving the ponto-medullary tegmentum, superior cerebellar peduncles, ventral midbrain, subthalamus, and inferior striatum. Diffusion restriction is indicative of intramyelin edema, and it selectively involves regions of the brain which are already myelinated at birth [9]. Kato et al. also reported three cases showing restricted diffusion at the brainstem, basal ganglia, thalamus, and deep white matter [19]. It follows that although there is a wide spectrum of brain anomalies associated with mutations in PIG family genes, the identification of similarities in the lesion distribution and appearance on MRI can guide the diagnostic process through a pattern recognition approach [20].

On the other hand, although having epileptic encephalopathy, these patients had a more delayed symptom onset, less extensive MRI alterations and a less severe clinical outcome compared to our case. In fact, in our patient, initially, brain MRI showed alterations very similar to those described by Tarailo-Graovac et al., but subsequently, the newborn developed a severe worsening of the cerebral findings with persistence of the previous alterations and additional extensive cortical and subcortical involvement of the cerebral hemispheres.

Interestingly, we can consider this evolution of brain alterations as two different patterns. The first “milder” pattern is characterized mainly by brainstem and deep gray matter involvement, and it has been described in the literature as part of the PIG genes deficiency-related spectrum [9]. The second “severe” pattern is characterized by diffuse cortical and subcortical involvement of brain hemispheres, and it has never been previously reported in this context.

Considering that our patient inherited two different mutations of the PIGO genes from both his parents, we can speculate that the combination of them led to a particularly severe clinical and neuroradiological phenotype, and this hypothesis is supported by the MRI findings. More specifically, we hypothesized that that the paternal and better-known mutation was correlated with the “milder” pattern, which was evident from the first MRI, while the maternal newly described mutation might have been responsible for the second “severe” pattern that we observed in the following MRI. It is necessary to point out that the “severe” pattern, characterized by extensive damage to cortical and subcortical brain tissue, has several differential diagnoses including hypoglycemia-related injury, hypoxic-ischemic encephalopathy (HIE), and hypernatremic dehydration-related injury [21,22,23]. All these differentials were considered in our case as well, but they were eventually ruled out in the clinic and laboratory.

## 4. Conclusions

We report the first case of compound heterozygosis of the PIGO gene, which has never been reported previously and may have been responsible for this peculiar neuroradiological picture, and the worse clinical outcome.

This hypothesis needs further validation through the analysis of more cases, but it may bring new insights on glycosylation-related disorders brain manifestations.

## Data Availability

The data are available from the corresponding author, F.D., upon reasonable request.

## References

[1] Wu T., Yin F., Guang S., He F., Yang L., Peng J. (2020). The Glycosylphosphatidylinositol biosynthesis pathway in human diseases. Orphanet J. Rare Dis..

[2] Starosta R.T., Kerashvili N., Pruitt C., Schultz M.J., Boyer S.W., Morava E., Lasio M.L.D., Grange D.K. (2023). PIGO-CDG: A case study with a new genotype, expansion of the phenotype, literature review, and nosological considerations. JIMD Rep..

[3] Sidpra J., Sudhakar S., Biswas A., Massey F., Turchetti V., Lau T., Cook E., Alvi J.R., Elbendary H.M., Jewell J.L. (2024). The clinical and genetic spectrum of inherited glycosylphosphatidylinositol deficiency disorders. Brain.

[4] Kuwayama R., Suzuki K., Nakamura J., Aizawa E., Yoshioka Y., Ikawa M., Nabatame S., Inoue K.I., Shimmyo Y., Ozono K. (2022). Establishment of mouse model of inherited PIGO deficiency and therapeutic potential of AAV-based gene therapy. Nat. Commun..

[5] Tanigawa J., Mimatsu H., Mizuno S., Okamoto N., Fukushi D., Tominaga K., Kidokoro H., Muramatsu Y., Nishi E., Nakamura S. (2017). Phenotype-genotype correlations of PIGO deficiency with variable phenotypes from infantile lethality to mild learning difficulties. Hum. Mutat..

[6] Brodsky R.A. (2014). Paroxysmal nocturnal hemoglobinuria. Blood.

[7] Hill A., DeZern A.E., Kinoshita T., Brodsky R.A. (2017). Paroxysmal nocturnal haemoglobinuria. Nat. Rev. Dis. Primers.

[8] Colden M.A., Kumar S., Munkhbileg B., Babushok D.V. (2022). Insights Into the Emergence of Paroxysmal Nocturnal Hemoglobinuria. Front. Immunol..

[9] Tarailo-Graovac M., Sinclair G., Stockler-Ipsiroglu S., Van Allen M., Rozmus J., Shyr C., Biancheri R., Oh T., Sayson B., Lafek M. (2015). The genotypic and phenotypic spectrum of PIGA deficiency. Orphanet J. Rare Dis..

[10] Nakamura K., Osaka H., Murakami Y., Anzai R., Nishiyama K., Kodera H., Nakashima M., Tsurusaki Y., Miyake N., Kinoshita T. (2014). PIGO mutations in intractable epilepsy and severe developmental delay with mild elevation of alkaline phosphatase levels. Epilepsia.

[11] Aguech A., Sfaihi L., Alila-Fersi O., Kolsi R., Tlili A., Kammoun T., Fendri A., Fakhfakh F. (2023). A novel homozygous PIGO mutation associated with severe infantile epileptic encephalopathy, profound developmental delay and psychomotor retardation: Structural and 3D modelling investigations and genotype-phenotype correlation. Metab. Brain Dis..

[12] Zehavi Y., von Renesse A., Daniel-Spiegel E., Sapir Y., Zalman L., Chervinsky I., Schuelke M., Straussberg R., Spiegel R. (2017). A homozygous PIGO mutation associated with severe infantile epileptic encephalopathy and corpus callosum hypoplasia, but normal alkaline phosphatase levels. Metab. Brain Dis..

[13] Krawitz P.M., Murakami Y., Hecht J., Krüger U., Holder S.E., Mortier G.R., Delle Chiaie B., De Baere E., Thompson M.D., Roscioli T. (2012). Mutations in PIGO, a member of the GPI-anchor-synthesis pathway, cause hyperphosphatasia with mental retardation. Am. J. Hum. Genet..

[14] Thompson M.D., Nezarati M.M., Gillessen-Kaesbach G., Meinecke P., Mendoza-Londono R., Mornet E., Brun-Heath I., Squarcioni C.P., Legeai-Mallet L., Munnich A. (2010). Hyperphosphatasia with seizures, neurologic deficit, and characteristic facial features: Five new patients with Mabry syndrome. Am. J. Med. Genet. A.

[15] Mabry C.C., Bautista A., Kirk R.F., Dubilier L.D., Braunstein H., Koepke J.A. (1970). Familial hyperphosphatase with mental retardation, seizures, and neurologic deficits. J. Pediatr..

[16] Brucker W.J., Croteau S.E., Prensner J.R., Cullion K., Heeney M.M., Lo J., McAlvin J.B., Peeler K., Shah N., Yee C.S.K. (2020). An emerging role for endothelial barrier support therapy for congenital disorders of glycosylation. J. Inherit. Metab. Dis..

[17] Briolay A., Bessueille L., Magne D. (2021). TNAP: A New Multitask Enzyme in Energy Metabolism. Int. J. Mol. Sci..

[18] Bayat A., Aledo-Serrano A., Gil-Nagel A., Korff C.M., Thomas A., Boßelmann C., Weber Y., Gardella E., Lund A.M., de Sain-van der Velden M.G.M. (2022). Pyridoxine or pyridoxal-5-phosphate treatment for seizures in glycosylphosphatidylinositol deficiency: A cohort study. Dev. Med. Child. Neurol..

[19] Kato M., Saitsu H., Murakami Y., Kikuchi K., Watanabe S., Iai M., Miya K., Matsuura R., Takayama R., Ohba C. (2014). PIGA mutations cause early-onset epileptic encephalopathies and distinctive features. Neurology.

[20] Van der Knaap M.S., Valk J., de Neeling N., Nauta J.J. (1991). Pattern recognition in magnetic resonance imaging of white matter disorders in children and young adults. Neuroradiology.

[21] Vyas S., Saini A.G., Kaur A., Singh P., Jayashree M., Sundaram V., Mukhopadhyay K., Singh P. (2021). Neuroimaging Spectrum of Severe Hypernatremia in Infants with Neurological Manifestations. Neuropediatrics.

[22] Gu M.H., Amanda F., Yuan T.M. (2019). Brain Injury in Neonatal Hypoglycemia: A Hospital-Based Cohort Study. Clin. Med. Insights Pediatr..

[23] Musapasaoglu H., Agildere A.M., Teksam M., Tarcan A., Gurakan B. (2008). Hypernatraemic dehydration in a neonate: Brain MRI findings. Br. J. Radiol..

